# Pilot randomised controlled trial of Weight Watchers® referral with or without dietitian-led group support for weight loss in women treated for breast cancer: the BRIGHT (BReast cancer weIGHT loss) trial

**DOI:** 10.1186/s40814-019-0405-x

**Published:** 2019-02-13

**Authors:** Rumana S. N. Newlands, Maria Ntessalen, Julia Clark, Shona Fielding, Pat Hoddinott, Steven D. Heys, Geraldine McNeill, Leone C. A. Craig

**Affiliations:** 1 Health Services Research Unit, Institute of Applied Health Sciences, School of Medicine, Medical Sciences and Nutrition, University of Aberdeen, 3rd floor Health Sciences Building, Foresterhill, Aberdeen, AB25 2ZD UK; 2 NHS Grampian Department of Nutrition and Dietetics, Aberdeen, UK; 3 Medical Statistics Team, Institute of Applied Health Sciences, School of Medicine and Dentistry, University of Aberdeen, Polwarth Building, Foresterhill, Aberdeen, AB25 2ZD UK; 4 Nursing Midwifery and Allied Health Professions Research Unit, University of Stirling, Stirling, FK9 4LA UK; 5 School of Medicine, Medical Sciences and Nutrition, University of Aberdeen, Polwarth Building, Foresterhill, Aberdeen, AB25 2ZD UK; 6 NHS Grampian, Scotland, UK; 7 Institute of Applied Health Sciences & The Rowett Institute, School of Medicine, Medical Sciences and Nutrition, University of Aberdeen, Polwarth Building, Foresterhill, Aberdeen, AB25 2ZD UK

**Keywords:** Breast cancer, Weight loss, Weight Watchers, Group support, Pilot trial, Feasibility

## Abstract

**Background:**

Being overweight or obese following breast cancer diagnosis can increase cancer recurrence and mortality, so effective interventions for weight loss in this group could enhance survival. A pilot randomised controlled trial was conducted to assess whether a weight loss programme comprising generic Weight Watchers® referral offered to women treated for breast cancer with or without additional breast cancer-tailored dietetic support is feasible and shows promise for improving weight and quality of life (QoL).

**Methods:**

Participants were randomly allocated to 3 groups: Weight Watchers® referral (for 12 sessions of meetings and digital tools) plus 5 breast cancer-tailored dietitian-led group support sessions (WW Plus: *n* = 14), Weight Watchers® referral only (WW: *n* = 16) or control (Weight Watchers® referral after 3 months, *n* = 15). Feasibility was assessed based on retention rate, recruitment and randomisation process, meeting attendance, suitability of the setting and outcome measurement tools, unintended consequences, cost and observations of the dietetic sessions. Outcomes were measured at 0, 3 (‘trial exit’) and 12 months post intervention.

**Results:**

The response rate to the invitation was 43% (140/327) of whom 58 were eligible and 45 (median age 61.0 years; body mass index 30.2 kg/m^2^) were randomised. Data from 38 (84%) and 30 (67%) participants were available at trial exit and 12 months respectively. Feasibility issues included slow recruitment process, lack of blinding throughout, weighing scales not measuring > 150 kg, lack of clear instructions for completing QoL questionnaire and workload and time pressures in delivering dietetic sessions. Participants had good attendance rate at group meetings and no serious unintended consequences were reported. WW Plus was most expensive to run. Mean (95% CI) weight change at trial exit was − 3.67 kg (− 5.67, − 2.07) in WW Plus, − 6.03 kg (− 7.61, − 4.44) in WW group and + 0.19 kg (− 1.45, + 1.83) in control group. About 40% of the WW Plus, 64% of the WW group and 56% of the control group lost ≥ 5% of their baseline weight by 12 months. All groups showed promise for improving QoL at trial exit but only the WW group maintained significant improvements from baseline at 12 months.

**Conclusions:**

The trial procedures were feasible, with some modifications. This pilot trial indicates the benefits of providing free WW vouchers for weight loss maintenance and improving QoL but provided no evidence that including additional dietetic support would add any extra value. Further research with WW with long-term follow-up should be undertaken to assess weight loss sustainability and benefit on health outcomes in this patient group.

**Trial registration:**

ISRCTN-29623418.

**Electronic supplementary material:**

The online version of this article (10.1186/s40814-019-0405-x) contains supplementary material, which is available to authorized users.

## Background

Breast cancer is the most common cancer among women worldwide, accounting for 25% of all female cancer cases and 12% of all cancer cases in 2012 [1, 2]. There are an estimated 1.67 million new cases of breast cancer and around 522,000 deaths from breast cancer each year [3]. The majority of women (50–96%) gain weight following breast cancer diagnosis and its treatments, with an average weight gain of 2.5–6.2 kg within 2 years of diagnosis [4–6]. Post-diagnosis weight gain is mainly associated with lifestyle changes (i.e. reduced energy expenditure and/or increased dietary intake) related to various treatment-related side-effects such as fatigue, change in sense of taste, psychological distress, functional impairments and poor quality of life (QoL) (Irwin, Melinda [53] L 2003) [4, 6, 8] (Maley, Mary 2013 [54]). In contrast, weight loss following breast cancer diagnosis is not as common as weight gain [7]. The weight gain pattern observed in women with breast cancer is much higher than healthy women in general in the United Kingdom (UK) population in whom, body mass index (BMI) increases on average about 0.1 BMI point (approximately 0.3 kg) per year [8, 9].

Being overweight or obese following diagnosis of breast cancer is associated with metabolic and hormonal profiles which may stimulate breast cancer growth in both pre- and post-menopausal survivors [10, 11]. Several systematic reviews and meta-analyses have found a significant association between weight gain of more than 5% and increased hazard of both breast cancer-related and all-cause mortality compared to women who maintain weight following diagnosis [10–12], with evidence of a dose-response relationship and a suggestion of a larger effect among women with a pre-diagnosis BMI < 25 kg/m^2^ [10]. Collectively, these findings suggest that efforts to minimise weight gain after diagnosis may improve survival [13]. Two large randomised controlled trials (RCTs) assessing the adoption of a diet that was either high in vegetables, fruit and fibre and low in fat (*n* = 3088, 7.3 year follow-up) (Pierce, John P 2007 [55]) or reduced fat (*n* = 2437, median 5-year follow-up) (RT, Chlebowski 2006 [56]), concluded that changes in dietary pattern alone (without any weight loss) is not sufficient to reduce additional breast cancer events or mortality in breast cancer survivors. Although the number of trials testing lifestyle interventions in breast cancer survivors is growing, it is still unknown whether intentional weight loss improves breast cancer-related outcomes (long-term disease-free survival, reduced overall mortality) in survivors [4, 14, 15].

There is no specific guidance for weight management in breast cancer survivors. Leading cancer organisations recommend that cancer survivors achieve and maintain a healthy body weight using similar strategies to those available for the general adult population [16, 17] with a combination of dietary, physical activity and behavioural strategies [18, 19]. The time after a cancer diagnosis has been described as a ‘teachable moment’ for health promotion such as weight management [20]. It could be that cancer patients are more motivated to engage in healthy lifestyle behaviours for a better prognosis [20]. However, previous research has found that adherence to recommended health behaviours for this population is less than optimal [21] with lack of support from the medical team, lack of knowledge regarding a healthy diet and its benefits, lack of access to available information and guidance, poor motivation, as well as various co-morbidities (e.g. functional impairment) cited as barriers to change [22–24]. Cancer survivors are a vulnerable population and weight loss can be extremely difficult due to the physical and mental challenges of cancer diagnosis and its treatment. Therefore, identifying and suggesting feasible weight loss components in women treated for breast cancer and investigating the impact of weight loss on longer-term prognosis is important [25].

The overall purpose of this study was to assess the feasibility and practicality of conducting a RCT of a weight loss programme in women treated for breast cancer and hence, replicating this trial to a definitive trial. The study was guided by the Medical Research Council (MRC) framework for developing and evaluating complex interventions [26]. Preparatory work involved a systematic review of RCTs targeting weight loss in women treated for breast cancer, synthesis of other relevant evidence (e.g. current recommendations for cancer survivors and the general population of adults for supporting weight loss-management) and a mixed methods study [focus group meetings (*n* = 15), survey (*n* = 139) and interviews (*n* = 20)] with the target population to understand their experiences and future preferences of a weight loss programme [27–30]. A summary of the findings of the preliminary study is presented in Additional file 1: Appendix 1.

Referral to Weight Watchers® (WW) were included as an intervention in order to address some of the modifiable barriers identified in the preliminary study, such as free access to a weight loss programme, education and social support for weight loss. The WW programme is based on the National Institute for Health and Care Excellence (NICE) guideline for obesity management and 14 various behaviour change techniques (BCTs) [31]. It has been found to be effective for clinically significant weight loss in the general adult population [32, 33] as well as in women treated for breast cancer [34], when free WW vouchers were given. In addition, a group-based programme similar to the format of many commercial weight management programmes was preferred by our preliminary study participants due to their flexibility (e.g. available at different times and days of the week, the option to attend a full session or drop in for getting weighed) and coaches have experienced a weight loss journey themselves. At the time of this study, WW referral was available through the UK primary care referral scheme to provide weight loss support to overweight and obese patients. The original plan was that the WW leaders would receive additional training from a dietitian and breast care nurses to deliver breast cancer-related extra support for breast cancer patients. However, WW would not allow any modification of WW regular group meeting contents and training of the leaders by anyone other than WW. As a result, a breast cancer-tailored dietetic-led group support programme was developed as an additional component to be delivered by a dietitian and a breast care nurse along with the WW programme to check its feasibility and whether it shows promise for greater weight loss and improvement in QoL compared to generic WW referral only or a control group. WW programme was the main component for weight loss and the dietetic-led sessions were aimed to provide social support through being with people who had experienced similar issues, encourage self-monitoring of lifestyle behaviour to maintain a healthy weight and improve knowledge by providing information related to diet, physical activity and healthy lifestyle.

This article reports the feasibility of the trial procedures and the outcomes related to participants in the pilot trial. A pilot trial is a smaller version of a future definitive trial in which all or some parts of the intervention to be evaluated and other processes (e.g. randomisation) to be undertaken in a future trial is/are carried out (piloted) to check its feasibility (Eldridge, Sandra M 2016 [57]). The research question asked in our pilot trial was: *Is conducting a weight loss programme comprising generic WW referral offered to women treated for breast cancer with or without additional breast cancer*-*tailored dietetic support feasible*? The primary outcome assessed was feasibility of the trial procedures from recruitment until trial exit. The specific objectives included assessing the facilitators and barriers towards conducting, assessing and/or delivering the trial components. The secondary outcomes assessed were changes in body weight and QoL for each group from their baseline.

## Methods

### Trial design

A pragmatic single-centre pilot parallel group randomised controlled trial RCT, involving three arms: WW Plus (Weight Watchers® referral to 12 sessions of community meetings and digital tools, plus 5 breast cancer-tailored dietitian-led group support), WW group (Weight Watchers® referral) and control group (Weight Watchers® referral after 3 months) (discussed under ‘Trial arms’ section), was designed to evaluate the whole process and inform decisions about whether a future full-scale RCT is feasible and shows promise.

This trial was conducted in the North East of Scotland between September 2013 and June 2015.

The trial was run in two cohorts: cohort 1 (November 2013–February 2013) and cohort 2 (March–June 2014) to avoid a long interval between recruitment, baseline meeting and trial entry. Changes in body weight and QoL were assessed at the trial exit which was week 14 (post randomisation) for the WW Plus group and week 12 (post randomisation) for the WW and control groups. The intervention period was longer for the WW Plus compared to other arms because participants attended two sessions of the dietetic-led group support programme before they received the WW referral pack for 12 sessions. An opportunity to follow-up participants at 12 months post intervention (March–June 2015) arose subsequent to the initial protocol. An amendment to the protocol was made which the ethics committee approved. There were no contacts with the research team until they were invited for further assessment at 12 months. It should be noted that there was lack of blinding for allocation, outcome assessment and analysis throughout the trial because this was a PhD project for a researcher (RN) who was the main contact with participants.

### Recruitment and group allocation

Women attending the Breast Clinic at Aberdeen Royal Infirmary (ARI, the main hospital in Aberdeen City) between September 2013 and January 2014 were sent an invitation letter from a senior breast cancer specialist (SDH) along with a patient information sheet and an opt-in form 2–3 weeks prior to their next scheduled appointment. Women who were interested in taking part in the trial sent the opt-in form back to the researcher (RN) in a postage paid envelope. Based on this opt in form, eligible participants were contacted to attend a baseline meeting.

The inclusion/exclusion criteria before randomisation are given in Table 1.

**Table 1:** Inclusion/exclusion criteria

Inclusion criteria	▪ Women who had completed initial treatment (surgery, chemotherapy and/or radiotherapy) for breast cancer
	▪ Age ≥ 18 years
	▪ Body mass index ≥ 25 kg/m^2^
Exclusion criteria	▪ Known distant metastases prior to study entry
	▪ Currently participating in any supervised weight loss programmes
	▪ Participated in Weight Watchers programme within the previous 3 months^a^
	▪ Pregnant women^a^
	▪ Diagnosed eating disorder^a^
	▪ Need interpreter to understand English

^a^Criteria suggested by the WW programme

Formal sample size power calculations were not carried out as this was a pilot study and the focus was not on formal testing of hypotheses but estimating parameters for a full-scale future trial [35]. It has been suggested that the sample of a pilot study should be representative of the target population and large enough to estimate parameters for a future trial [35] (Shanyinde M. 2011 [58]). The initial target was to recruit 30 participants per arm as the Chief Scientist Office (Scottish Government Health Directorates) guidelines for developing pilot studies suggests (Chief Scientist Office 2014 [59]). The opt-in form asked potential participants to provide information on their current weight and height, year of diagnosis, recent participation in commercial weight management programmes and possibility of pregnancy, to assess their eligibility.

#### Baseline meeting

Potential participants were invited to attend a one-to-one baseline meeting with the researcher (RN) at the Maggie’s Cancer Support Centre located within the ARI health campus; an attractive setting for its sensory aspects with easy access to facilities (i.e. regular public transport and parking spaces for the centre’s use). Reimbursement for travel expenses incurred by participating in the trial was offered to all participants. At the baseline meeting, participants who agreed to take part were asked to give their informed consent (written) and, for consenting participants, their height, weight and QoL were then measured to confirm eligibility based on BMI and so that randomisation could be carried out with minimisation of the differences in BMI between groups. These methods were used to avoid recruiting participants with a ≤ healthy BMI and/or to minimise differences between groups. We learned from previous work that often patients could not remember their current height and weight or reported a weight and/or height that were taken a long time ago [47, 48]. Height was measured to the nearest 0.1 cm with a Leicester portable height measure (Marsden, UK), without shoes and with the back straight and the head in the Frankfurt plane. Weight was measured to the nearest 0.1 kg with electronic scales (SECA, Model 803, Hamburg, Germany) with participants wearing light clothing and no shoes. BMI (kg/m^2^) was calculated as weight (kg) / height (m)^2^.

The Functional Assessment of Cancer Therapy-Breast (FACT-B) questionnaire (version 4) was used to assess QoL as it is a validated instrument for cancer survivors and has also shown good sensitivity to change [36, 37]. It has a 5-point Likert-type response scale for 36-item measures that cover 4 Functional Assessment of Cancer Therapy-General (FACT-G) subscales: Physical Well-Being (PWB), Social/Family Well-Being (SWB), Emotional Well-Being (EWB) and Functional Well-Being (FWB) along with an additional Breast Cancer-Specific (BCS) subscale. The BCS subscale assesses symptoms/concerns of particular relevance to breast cancer (e.g. body image, arm swelling and tenderness).

#### Group allocation

Following the baseline meeting, each participant was randomised on a 1:1:1 ratio by a researcher (RN) using an in-house computer programme which allocated participants minimising differences between the groups in terms of three categorical variables: baseline BMI (< 30/≥ 30 kg/m^2^), age (< 60/≥ 60 years) and time since diagnosis (< 1/≥ 1 year). Participants were contacted by telephone (by a researcher, RN) to inform them of their group allocation and arrange subsequent meetings.

#### Trial arms

##### WW Plus (Weight Watchers referral plus breast cancer-tailored dietitian-led group support)

Participants were invited to attend five breast cancer-tailored dietitian-led support group sessions at the Maggie’s cancer support centre, Aberdeen over the course of 14 weeks. The sessions lasted 1–1.5 h and were in groups of 5–9 participants led by a research dietitian (JC: age group 40–50 years; British-white; female; registered dietitian) and a breast care nurse (age group 40–50 years; British-white; female; registered specialist nurse). The sessions involved PowerPoint presentations using scripts, displays of various food models and informal learning and discussion sessions. As both providers had experience in running group sessions involving patients, they were not provided with any specific training. A researcher (RN) was present in all meeting sessions as an observer and a facilitator (e.g. taking field notes, serving refreshments). Neither the group providers nor the participants were blinded to group allocation.

All materials and discussion topics were generated by JC based on the findings from the pre-design study along with her own expert dietetic knowledge and skills and discussion with the research team and stakeholders (three breast care specialist nurses). The contents of the presentation provided by the breast care nurse was generated and adapted from their previous materials to fit with the study purpose. Details of the content of these sessions are given in Additional file 1: Appendix 2.

At the second group meeting (week 2), participants were given a WW referral pack which included 12 free vouchers to community meetings plus 16 weeks’ access to digital content and tools. They were asked to join a suitable WW group at any location convenient to them as soon as possible, with other participants from the group as ‘buddies’ for social support if they wished. It should be noted that WW referral packs were bought by the research team directly from the WW and WW team was not involved in data collection, analysis or interpretation of the research findings.

##### Overview of the Weight Watchers programme

This programme recommends adoption of energy deficit or reduced energy diets high in fruit and vegetables and low in carbohydrates, dairy products and fat for weight loss maintenance. It also provides a guide for a range of diets (e.g. vegetarians, gluten free and vegan) and recipes including a variety of ethnic cuisines (e.g. Indian, Thai, Italian, American) and fast foods (Weight Watchers® 2015 [71]). Anyone can attend WW community meetings at any group and any location throughout the country. One-to-one support from the group coach is available during a weekly weighing session which is usually followed by a 20-min group discussion, which is curriculum based. Meetings take place in community-based venues (e.g. church hall, hotel or sports centres) and the whole session usually lasts for approximately an hour depending on the size of the group. Moreover, moderated social media groups, 24/7 live online support with a coach through expert chat, weekly inspiring content, e-mails with success stories and contact numbers of the group coach are provided for continuous support and motivation for lifestyle change. Core programme materials are given at the weekly meeting which include recipes, and tips for healthy nutrition and keeping active. Physical activity is encouraged throughout the programme. The weight loss system at the time of this pilot study was the ProPoints® weight loss system, which enables people to create an energy deficit for healthy weight loss using a points-based system; giving people a personalised budget to spend, with all foods and drinks having a value.

The free vouchers provided in the referral pack were processed at the WW meeting by the WW coach, who had not been informed of the pilot study. WW coaches supported participants as all other participants, including those who would come in via the ‘referral’ scheme. BRIGHT trial participants were told not to inform about their purpose of attending WW (i.e. trial participants) to the coach or other attendees at the WW meetings during the trial period.

##### WW group (Weight Watchers referral)

All participants in this group were sent the WW referral pack (12 free vouchers for access to community meetings plus 16 weeks’ access to digital content and tools) by post and asked to join the programme at a location convenient to them within 2 weeks of receiving the referral pack and contact the researcher (RN) to inform of their joining date. Participants had no other contact with the research team until the trial exit visit.

##### Control group

The participants did not receive any intervention, but at the trial exit (12 weeks following randomisation) each participant was given the WW referral pack to use whenever they wished.

### Outcomes

#### Primary outcomes

Primary outcome data were evaluated according to whether there were any issues that would affect the feasibility of conducting a future full trial: high retention rate (ideally > 80% as suggested by previous literature [38, 35]; no issues with the recruitment (i.e. the process would identify, invite and recruit the target number of potential participants in a straightforward manner), randomisation process (i.e. the programme would meet the purpose of group allocation), the setting for data collection and delivery of the intervention (i.e. the venue would be practical for the delivery of intervention and/or data collection) and the outcome measuring tools (i.e. tools would be reasonable for the purpose); no barriers towards adherence to the contents or delivery of dietetic-led group sessions (i.e. no substantial modifications to the protocol would be required); no serious unintended consequences (i.e. no hospitalisation, life-threatening condition or death associated with the intervention); good meeting attendance rate (≥ 50% as suggested by other weight loss studies with 12-week follow-up [32, 33, 70] and low cost. There were no pre-specified set of criteria to assess all the indicators. The aim was to evaluate any issues identified in this pilot and hence, propose solutions if modifiable, for any future trial.

#### Secondary outcomes

At trial exit, participants’ weight was measured by a researcher (RN) and they completed the FACT-B questionnaire at the Maggie’s Centre. At 12 months, all participants who had completed the trial were sent a letter along with a brief summary of the trial results inviting them to a one-to-one meeting with the study dietitian (JC) at the Maggie’s Centre. At this meeting, their weight was measured by a researcher (MN) and they completed the FACT-B questionnaire. They also completed an additional short questionnaire (available on request from the researchers) which asked questions on the use of the WW vouchers (How many WW vouchers provided in the study did you use? Have you attended any more WW meetings since finishing the vouchers provided in the study? Have you been following the WW ProPoints system or any other kind of weight loss programme since finishing the BRIGHT study?) and other lifestyle changes including dietary habits (Have you made any changes to your diet since finishing the BRIGHT study?) and physical activity (Have you changed your physical activity in any way since finishing the BRIGHT study?).

#### Data collection, management and analysis

All primary outcome data (quantitative and qualitative) were documented from beginning until trial exit in the form of field notes: recruitment process (i.e. duration of the process, time taken to screen and identify potential eligible patients using medical notes, any challenges experienced such as availability of the data to assess eligibility), randomisation process (i.e. whether the randomisation procedure was appropriate for the purpose, any identified issues), unintended consequences (i.e. any changes in the processes and/or any incidences that might impact on the delivery and outcomes of the trial), issues with the setting (i.e. barriers and facilitators of using the Maggie’s Centre for the baseline meeting and delivery of the intervention) and outcome measurement tools (i.e. whether the weighing scales, and FACT-B were acceptable for the purpose and any issues with outcome measurements). Adherence to content and competence in delivering the dietetic-led sessions was measured and rated as ‘yes’ or ‘no’, by a researcher (RN) during the group observations, depending on whether the group leader performed the expected action during that group session (i.e. whether the leader adhered to the contents of the sessions as planned and participants had the chance to ask questions) (Additional file 1: Appendix 3). Participants’ attendance at each dietetic-led session was recorded by the researcher (RN) at the beginning of each session. Participants in WW Plus and WW group were requested to self-report their attendance at the WW meetings to the research team at trial exit. The basic cost of the programme was calculated as an average per participant including cost of postage, WW referral pack and dietitian’s and breast care nurse’s time. Views of the intervention providers (dietitian and breast care nurses) were captured through one to one discussion using structured questions [How did you incorporate the BRIGHT trial work into your work routine? What barriers have you experienced during delivery of the group sessions?] and field notes.

All field notes were initially handwritten and then typed into a Microsoft Word document (including who, when, where, how and by whom as appropriate) and kept on a secure server at the University of Aberdeen. All qualitative data (i.e. field notes from recruitment to trial entry and discussions with the dietitian and breast care nurses) was analysed by the researcher (RN) using narrative analysis to interpret ‘what happened’ in relation to the feasibility of the trial procedures. This method of analysis is simple and is concerned with understanding ‘how and why’ any situation occurs to explain the entire process systematically (Lieblich, Amia 1998 [60]).

Most of the analysis was conducted using IBM SPSS Statistics version 22.0 and carried out by researchers (RN, MN and SF). Although hypothesis testing in this type of trial is not recommended, these tests were performed and confidence interval values reported in order to comment on the promise of the results and inform future sample size calculations. All tests were two-tailed, and significance was set at *p* < 0.05. Baseline characteristics (age, weight, BMI and time since diagnosis) were described using median and interquartile range in each group as these variables were not normally distributed. A target weight loss of ≥ 5% from baseline weight within 6 months for weight loss programmes is recommended, as it may lead to reduction in chronic disease risk such as diabetes and cardiovascular disease [39, 40]. Therefore, the proportion of participants who had lost 5% or more of their initial body weight at 12 months was calculated. Scoring of the QoL items was carried out in SPSS using a syntax file provided by the FACIT organisation (http://www.facit.org).

Linear mixed effects models were used to assess the change in weight for each group utilising data from baseline, 3 and 12 months. This part of the analysis was conducted by SF using SAS, version 9.3 (SAS Institute Inc., Cary, NC, USA). The model allows for missing data under the missing at random assumption as long as at least one outcome value is available. These models were fitted with an unstructured covariance with fixed effects of time, group and a time-group interaction. The same approach was used to model changes in each of the quality of life domains. All models were adjusted for BMI (< 30, ≥ 30 kg/m^2^), age at baseline (< 60, ≥ 60 years) and years since diagnosis (< 1, ≥ 1 year). Estimates of mean change (and 95% CI) at 3 and 12 months from baseline were calculated along with estimates of difference in these changes between groups.

## Results

### Primary outcomes

#### Feasibility of the procedures

Table 2 provides an overview of the findings of the trial procedures.

**Table 2:** Overview of the findings from the feasibility assessment of the trial procedures

Feasibility indicator	What did work well?	What did not work well? How to address these issues in the future (if modifiable)?
Retention	This study had a good retention rate (84%).	_
Recruitment	Staff at the clinic were co-operative and allowed researchers to screen notes in their busy working atmosphere and to use their system for printing labels (names and addresses of the potential participants) which helped to speed up the process.	The process could not meet the target of recruiting 30 women per arm in 4 month time scale within the resources available. *Solution:* More time would be required to achieve target number of participants if the same procedure were to be used in a future trial. Reminders could be sent to the non-responders. The actual response rate of eligible women to the invitation letter is unknown as information on BMI was not available in the clinic notes. *Solution:* Weight measurements made at out-patient clinics should be entered in clinic notes.
Randomisation	The overall group allocation process worked well.	Median years since diagnosis reported was 2.0 years but the allocation process used < 1 vs > 1 year since diagnosis. *Solution:* A future trial should use median time since diagnosis (e.g. 2 years) for group allocation.
Unintended consequences	No serious adverse events were recorded.	Two participants were distressed and one withdrew due to the setting and topics discussed. *Solution:* Venue needs to be carefully chosen and topics related to sensitive issues should be avoided.
Setting	Maggie’s centre was feasible for conducting baseline and end-point meetings and convenient for health professionals to deliver the dietetic led sessions.	There were problems with using slides in the dietitian-led group sessions as the room had no blinds or projector screen. There was a lack of parking space for the participants. *Solution:* Issues related to room setting could be addressed with portable equipment. Another non-hospital venue such as the CLAN cancer support centre or a community centre could be used in the future to avoid parking issues.
Data collection tools	The height measure and weighing scales were easy to use. The FACT-B QoL questionnaire did not take long to complete by participants.	The weighing scale could not measure weight above 150 kg. *Solution:* Scales with higher weighing capacity could be used. Some questions of the QoL questionnaire were felt by participants not to be applicable after a few years of finishing breast cancer treatments. *Solution:* FACT-B could be used with more clear instructions to avoid missing data or a different QoL questionnaire could be used.
Fidelity/delivery of the dietetic-led sessions	All facilitators adhered to the study protocol.	Workload and time were recorded as issues for delivering dietitian led group sessions. *Solutions:* Non-health professionals or volunteers could be trained to deliver the group sessions. It was not possible to observe WW sessions to report their fidelity. *Solution:* Random sessions could be observed or recorded to report fidelity. Also see issues discussed above under setting.
Meeting attendance	Good attendance rates at dietetic led sessions (85%) and WW programme (WW Plus = 78% and WW group = 68%).	Attendance data at WW programme was provided by participants. *Solution:* Participants’ booklet provided by WW could be assessed for accurate recording of attendance.
Cost	WW group was not expensive to run.	WW Plus group was expensive to run compared to WW group. *Solution:* Non-health professionals or volunteers can be trained to deliver the group sessions, which can be tested before inclusion. But then there might not be as good adherence and fidelity of delivery as observed when ran by a dietitian.

##### Recruitment

**Fig. 1:**
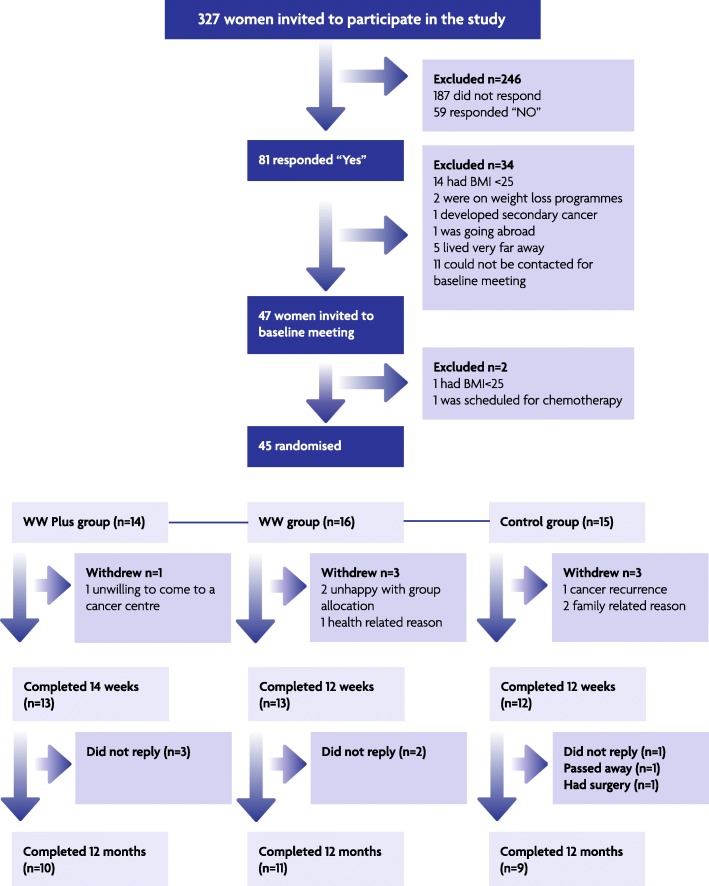
Flow chart showing recruitment and drop-outs at all stages of the pilot BRIGHT trial

Recruitment by one researcher (RN) at one hospital breast cancer out-patient clinic took 4 months (e.g. it took 4 h to screen 50 clinic notes where only 10 patients were eligible). The response rate to the invitation letter was 43% (140/327) of whom 25% (*n* = 81) were willing to participate and 18% (*n* = 58) were eligible (Fig. 1). Information on BMI was not available in the clinic notes so women with BMI < 25 kg/m^2^ also received the letter and some may not have replied as they knew that they would not be eligible. Therefore, we do not know the number of eligible women for this trial and hence the actual response rate of eligible women to the invitation letter is unknown. At the beginning of the recruitment, a few women with benign tumours were invited as it was not possible to identify the potential women from the clinic list. Consequently, following the first cohort of invitations, to avoid such issues, approval from the Caldicott Guardian (Medical Director, National Health Services, Grampian) was sought for the researcher (RN) to access medical notes to identify potentially eligible women.

Twenty-five of the 59 women who did not want to take part in the trial provided reasons for non-participation: benign breast disease (*n* = 8), living too far from Aberdeen to attend group meetings (*n* = 7), currently following other weight loss programmes (*n* = 2), recently lost weight (*n* = 2), diagnosed with dementia (*n* = 2) or secondary cancer (*n* = 1), not interested in WW (*n* = 1), felt they were too old (*n* = 1) or working night shifts (*n* = 1).

##### Baseline characteristics

Forty-seven out of the 58 eligible women (81%) were invited to and attended the baseline meeting. The remaining 11 women could not be contacted (e.g. not available to answer a telephone call, no reply to email or no option to leave a voicemail). Two of the 47 potential participants were found to be ineligible following the baseline meeting as one had a healthy weight when measured and another was scheduled to receive chemotherapy.

The median (IQR) age of the participants at recruitment was 61.0 (53.5, 67.0) years with 51% of participants being over 60 years. Median (IQR) BMI was 30.2 (27.4, 32.7) kg/m^2^ with 53% of participants having a BMI over 30 kg/m^2^ (Table 3). The median (IQR) time since cancer diagnosis was 2.0 (1.0, 4.5) years with all but one of the participants entering the trial more than 1 year after their diagnosis. Forty-four participants were White, British and 1 was African British.

**Table 3:** Baseline characteristics of participants

Variable/Characteristic	All *n* = 45	WW Plus group *n* = 14	WW group *n* = 16	Control group *n* = 15
Age at baseline (years)				
Median [IQR (25th, 75th)]	61.0 (53.5, 67.0)	60.0 (53.7, 67.5)	60.0 (51.0, 66.0)	61.0 (52.0, 70.1)
Menopausal status at diagnosis (%)				
Pre-menopausal	35.6%	28.6%	43.8%	33.3%
Post-menopausal	64.4%	71.4%	56.3%	66.7%
Time since cancer diagnosis^a^ (years)				
Median [IQR (25th, 75th)]	2.0 (1.0, 4.5)	2.0 (1.8, 5.0)	2.0 (1.0, 3.8)	2.5 (1.0, 5.0)
Baseline weight (kg)				
Median [IQR (25th, 75th)]	76.6 (70.9, 85.1)	76.6 (69.9, 85.2)	77.2 (72.6, 85.3)	76.6 (70.2, 89.5)
BMI at baseline (kg/m^2^)				
Median [IQR (25th, 75th)]	30.2 (27.4, 32.7)	30.1 (27.0,33.1)	30.4 (27.7, 32.9)	30.2 (26.5, 33.1)

*IQR* interquartile range

^a^Time interval between breast cancer diagnosis and study entry

##### Randomisation

A total of 45 participants were allocated to the 3 arms: 14 to WW Plus group, 16 to WW group and 15 to control group. The randomisation procedure used was feasible for the purpose, but it became apparent the cut-off for years since diagnosis needs to be amended for future studies. With a median of 2 years, the suggestion would be < 2/≥ 2 years instead of the < 1/≥ 1 we used here.

Cohort 1 involved 17 participants (WW Plus = 5, WW group = 6 and control group = 6) and cohort 2 involved 28 participants (WW Plus = 9, WW = 10 and control group = 9).

##### Retention

Follow-up data (primary and secondary outcomes) were available for 38 participants [84%; WW Plus = 13 (93%), WW group = 13 (81%) and control group = 12 (80%)] at trial exit. Reasons for missing outcome data were unhappy with the allocated group (WW group = 2), health issues unrelated to cancer (WW group = 1), recurrence of cancer (control group = 1), family-related reason [e.g. lost partner (control group = 2)] and did not want to attend group sessions in a cancer support centre (WW Plus = 1). In total, data from 30 participants [67% of trial completers (WW Plus = 10, WW group = 11 and control group = 9)] were available at 12 months post intervention follow-up. Of which, 27/30 women attended the one-to-one follow-up meeting and 3 provided measurements by post. Reasons for missing data were passed away (control group = 1), had surgery (control group = 1) and did not reply to the invitation (WW Plus = 3; WW group = 2 and control group = 1).

##### Attendance

Twelve of the 14 (85%) participants in WW Plus group attended all 5 group meetings. Data on attendance at the WW meeting was provided by the participants. Eleven participants from WW Plus group (78%) and 11 participants from WW group (68%) reported attending all 12 WW meetings.

##### Adherence

At 12 months follow-up, participants from the WW group (46%, 5/11) reported having attended WW meetings more often than WW Plus (30%, 3/10) or control group (25%, 2/8) since finishing the referral provided in the trial. Seventy percent (7/10) of the WW Plus, 64% (7/11) of the WW group and 38% (3/8) of the control group participants reported continuing to follow the WW ProPoints weight loss system as well as improving their physical activity since finishing the BRIGHT trial. Finally, 50% (5/10) of the WW Plus, 64% (7/11) of the WW group and 52% (4/7) of the control group participants reported having made some other changes in their diet including reduction of portion size, eating more fruits and vegetables and reduction of the amount of ‘fattening’ foods following trial exit.

##### Setting

A private room was used for all one-to-one meetings. The staff members at the Maggie’s Centre were very welcoming and helpful. An advantage of using this setting is that refreshments were offered to the participants on their arrival, and if participants arrived earlier than the scheduled time or if the researcher was busy with another participant, they were kept company by a member of staff. There were some issues with the setting of the room for delivering the dietetic-led sessions: no blinds in the room, which affected the visibility of the projector screen and hence the room had to be re-arranged by the research team to avoid this issue. There was no projector or screen on site available to use, so the equipment was borrowed from the University for delivering the sessions; the projector was kept on top of a table and wires were lying on the floor. Although this was not ideal, we tried our best to maintain health and safety standards. Although the centre had their own parking, it was sometimes not sufficient when there were other sessions/meetings at the same time as our meetings or other hospital patients parked at the Maggie’s allocated parking spaces. As a result, on a few occasions participants were late for their appointments.

##### Delivery of the intervention (observation of dietetic-led sessions from researcher)

The first meeting started with the introduction of the study and team members involved in the whole programme. Some participants attended the meetings with their partners who socialised with other partners in the Maggie’s centre while the women took part in the meetings. Participants discussed their worries about the weight loss journey due to their failure of achieving healthy weight following diagnosis. They mentioned that it was fun answering the true/false questions, e.g. potatoes belong to the fruit and veg section (true or false?). Most of them were surprised that potato was classed as a starchy food and not a vegetable. Most of them were also unaware of the amount of calories hidden in their favourite drinks. They were engaged with the facilitators and contributed their knowledge and habits in relation to the topics discussed. Details of the content of these sessions are available in Additional file 1: Appendix 2.

The second meeting started with a talk by the breast care nurse followed by the last half session on physical activity being led by the dietitian (JC). During the first half session, participants laughed relating their own experiences with the topics discussed and agreed that they had experienced most of the symptoms, e.g. increased appetite, altered taste and less activity. One participant was emotionally distressed during the discussion of changes in body image (i.e. due to hair loss and/or mastectomy) and  relationships and left the room for a short period of time. It was mentioned that she felt distressed during the discussion about relationship issues as it reminded her about her personal experiences at the time of receiving treatments. Participants mentioned that this type of information (i.e. tips for maintaining a positive body image, prosthesis/underwear) would have been helpful soon after diagnosis or finishing treatment, as the information received during treatment was too much to take in. During the physical activity talk, participants were fully engaged and agreed that 30 min of physical activity out of 1440 min a day is easily achievable with self-motivation. At the end of this session, on receiving the WW vouchers, participants looked very happy and excited to start something new.

At the beginning of the third meeting, participants discussed their experience of the WW meetings with other group members and the dietitian (JC). Some took their folder, leaflet and recipes provided by WW to this meeting and realised that they had all received different resources in the same week as they all attended different WW meetings. One participant mentioned that she felt she was being treated differently by the WW leader as she was given a red folder and the rest of the members of WW had a blue folder, and the reason given by the WW leader was ‘you are special’. During the discussion, it emerged that all participants (from our study) had received a red folder signifying participation in a referral pack scheme.

This meeting covered topics related to food labels, recommended portion sizes, calorie counting and tips for portion control. Most participants mentioned that different supermarkets use different labels and they experienced that these labels are difficult to read and understand. Therefore, discussion on labels (i.e. colour-coded information on how to read a label) for similar type of products were found to be very useful. Most participants agreed regarding the reasons discussed why people eat more than they need (e.g. habit, satisfaction, taste, reluctant to waste food and larger restaurant portions) and mentioned that tips for eating less (e.g. listen to hunger; if a portion is large share with a friend) would help them to change habits. However, some said that they already knew most of these topics being discussed, but they had never had the motivation to follow these to change to a healthy lifestyle.

During the fourth meeting, food models were displayed on two tables for the participants to see and discuss. The dietitian (JC) started the session by asking how they were getting on with WW and recapping the previous three talks. All participants in cohort 1 had 2 weeks off from WW during Christmas time as no classes were run over that period, and they all mentioned that they found it very difficult to lose weight during that time. The dietitian (JC) gave a talk on hidden calories (topics covered various names of sugars to identify in food labels such as fructose; high vs. low fat foods) and how to avoid these calories (e.g. changing to semi-skimmed milk could save 60 cal a day or 420 cal a week) followed by looking at the food models and discussion. During the slide show on fats, some mentioned swapping cooking ingredients with healthy options such as margarine and non-creamy sauce, although some mentioned not liking the taste. One participant said that she thought gin and tonic had fewer calories than a glass of wine and was surprised at seeing the model which showed that both contain similar calories. They were also shocked to find out that Heinz tomato soup contains a lot of sugar.

During the last meeting, participants were asked about their whole experience of the last four meetings and if they had any queries related to diet, physical activity and weight loss. They discussed how much they had lost so far based on the assessment by WW and how they were managing their lifestyle. Most participants mentioned that they tried to follow a healthy lifestyle based on what they have learnt from the dietetic-led group meetings rather than strictly following the WW ProPoints® system suggested by the WW programme. They also discussed that if their weight was assessed in every group meeting by the dietitian (JC), they would have been more motivated to lose the weight. They mentioned that the quiz session was fun and it captured their understanding of the previous sessions. They also enjoyed and had a laugh together when JC disclosed the right answers to the quiz questions. The session ended by thanking the participants for taking part in the trial and a plant was given to each participant and flower bouquets to the 1st, 2nd and 3rd prize winners of the quiz. All participants expressed enjoying every session and being disappointed that the trial had concluded.

Qualitative data collected confirmed the fidelity in that the trial was delivered as intended by the researchers and also it adhered to the study protocol including duration of contact time (length of time of intervention period), and intervention contents. No modifications in trial procedures were required for cohort 1 or 2. The dietitian and breast care nurses had adequate expertise and skills to answer participants’ queries and make the sessions enjoyable.

It was not possible to observe WW sessions to report their fidelity due to the different timings and locations at which our participants attended. However, the organisation have a matrix of QA and audit in place to oversee fidelity of programme delivery in the network of 6000 meetings in the UK. However, these data were not made available to our research team.

Both facilitators (dietitian and breast care nurse) discussed how they had incorporated this trial work into their existing workloads. As an example, the breast care nurse team required advanced notice (e.g. minimum of 2 weeks) of the scheduled day and time to take time out of their routine job to deliver the session. It might be a problem for them to deliver the intervention out of working hours such as evenings or weekends.

##### Outcome measurement tools

The height measure and the weighing scale were easy to use. The weighing scale could not measure weight above 150 kg and hence one participant had to be taken to a different building to measure her weight on a suitable scale. In relation to the FACT-B questionnaire, it took on average 8 min to complete the questionnaire by the participants. Participants had no problem filling in the questionnaire. There were very few missing data for the questions except for ‘I feel sexually attractive’ (*n* = 2), ‘I feel close to my partner (or the person who is my main support)’ (*n* = 2), ‘I am bothered by side-effects of treatment’ (*n* = 1) and ‘I am bothered by hair loss’ (*n* = 1). The latter two questions were thought to be not applicable after a few years of finishing breast cancer treatments. However, 12 participants missed out the question ‘I am satisfied with my sex life’ as it was felt to be not applicable—but this was not a problem as there was an option to skip this question and go to the next section. The researcher had to read out the questions word for word for three participants due to issues with poor eyesight and/or forgetting reading glasses.

##### Cost

WW Plus group (£167.72 per participant) was more expensive to run than the WW group (£59.31 per participant) or control group (£57.72 per participant), due to the additional cost of the facilitators to run the dietitian-led sessions.

##### Unintended consequences

Only two unintended consequences were recorded: one participant from WW Plus group did not want to continue participating in the trial as the cancer support centre and topics discussed in the dietetic-led group meeting related to ‘cancer and relationship issues’ had reminded her of cancer diagnosis. Another participant also from the WW Plus group was upset during the talk on changes in body image and relationships (discussed earlier) but remained in the trial. Discussion on sensitive issues, if unrelated to trial aim and objectives such as issues with hair loss or relationship, should be avoided.

#### Secondary outcomes

##### Changes in body weight

Table 4 describes the results of the mixed effects model. Both WW Plus and WW group showed significant (*p* < 0.001) weight change from baseline to 3 months, but the control group did not (Table 4). Around 58% participants in WW group and 37% in WW Plus group achieved clinically important ≥ 5% loss of initial weight at trial exit. The change in weight from baseline to 12 months was significant for the control group (− 4.22 kg, received WW referral following trial exit) and the WW group (− 5.11 kg), but was no longer significant for the WW Plus group (− 1.22 kg).

**Table 4:** Mean weight loss over 12 months after fitting a linear mixed model

	Weight change over 12 months		
	WW Plus group *n* = 14	WW group *n* = 16	Control group *n* = 15
Baseline—3 months			
Weight change (95% CI) (kg)	− 3.67 (− 5.67, − 2.07)	− 6.03 (− 7.61, − 4.44)	+ 0.19 (− 1.45, 1.83)
*p* value	*p* < 0.001	*p* < 0.001	*p* = 0.815
Baseline—12 months			
Weight change (95% CI) (kg)	− 1.22 (− 4.42, 1.98)	− 5.11 (− 8.18, − 2.05)	− 4.22 (− 7.55, − 0.88)
*p* value	*p* = 0.436	*p* = 0.002	*p* = 0.015

However, 40% of the WW Plus group, 64% of the WW group and 56% of the control group lost ≥ 5% of their baseline weight by 12 months. There were no significant differences found in weight changes in any of the intervention arms (WW Plus, *p* = 0.536; WW group, *p* = 0.240 or control group, *p* = 0.522) between batch 1 vs. batch 2.

##### Changes in QoL

At trial exit and 12 months follow-up, in the adjusted model, all three groups showed statistically significant improvements from baseline in various subscales of the FACT-B (Table 5). The WW Plus group showed statistically significant improvements at trial exit on the Functional Well-being (FWB, *p* = 0.023), Functional Assessment of Cancer Therapy-General (FACT-G, *p* = 0.020), Breast Cancer Specific (BCS, *p* < 0.001) subscales, Total Trial Outcome Index (TOI, *p* = 0.003) and the overall scale of FACT-B (*p* = 0.002). The WW group showed statistically significant improvements at trial exit in Physical Well-being (PWB, *p* = 0.001), Emotional Well-being (EWB, *p* = 0.038), FACT-G (*p* = 0.006), BCS (*p* < 0.001) and TOI (*p* < 0001) sub-scales, while the control group showed statistically significant improvements at trial exit in the FACT-G (*p* = 0.023) and overall FACT-B (*p* = 0.026). At 12 months follow-up, the WW group showed statistically significant improvements from baseline on the PWB (*p* = 0.029), BCS (*p* = 0.008) and TOI (*p* = 0.006) sub-scales.

**Table 5:**
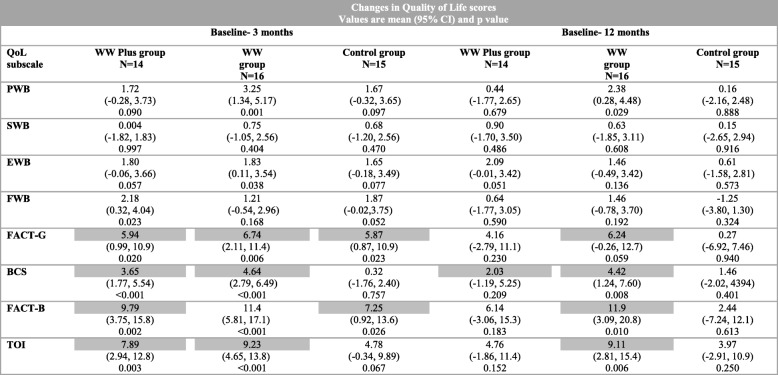
Changes in Quality of Life scores

Table showing changes in QoL scores between baseline and 12 months (linear mixed model) for Physical well-being (PWB), Social/Family well-being (SWB), Emotional well-being (EWB), Functional well-being (FWB), Functional Assessment of Cancer Therapy–General (FACT-G = PWB + SWB + EWB + FWB) and Breast Cancer specific (BCS) subscales, as well as the overall scale of Functional Assessment of Cancer Therapy—for breast cancer (FACT-B = FACT-G + BCS) and the Trial outcome index (TOI = PWB + FWB + BCS)

A higher value indicates greater improvement. Suggested minimally important differences (MID) for the scale: BCS = 2–3 points, TOI = 5–6 points, FACT-G total = 5–6 points and FACT-Breast total = 7–8 points (Eton, D.T. 2004 [61]). The values shaded in grey highlighting the subscales that obtained MID

A minimally important difference (MID) on a QoL measure corresponds to the smallest difference in score in the domain of interest that patients perceive as important, and which would lead the clinician to consider a change in patient’s management (Eton, D.T. 2004 [61]). The suggested MID for the FACT-B scale are BCS = 2–3 points, TOI = 5–6 points, FACT-G total = 5–6 points and FACT-Breast total = 7–8 points (Eton, D.T. 2004 [61]) (Table 5). At 12 months, the WW group achieved and maintained the MID in FACT-G, BCS, FACT-B and TOI, whereas WW Plus maintained it for BCS only.

## Discussion

Overall, this pilot trial suggested that a larger trial would be feasible as the retention rate was ≥ 80% and meeting attendance rate was ≥ 50%; no serious adverse events were recorded; and providing WW vouchers incurred low cost. The secondary outcomes also showed promise to support a larger, definitive trial. Suggested modifications to any future trial include assuring adequate blinding throughout the process, the cut-off for years since diagnosis should be < 2/≥ 2 years for the randomisation process, a weighing scale with higher weighing capacity should be used, and clear instructions for completing FACT-B should be provided to avoid missing data. If the same setting is used in the future, the issues related to delivering the interventions need to be addressed. Due to resource limitations, the recruitment was limited to one centre in Aberdeen and the target sample size could not be achieved in the available time. More time or additional centres would be required to recruit sufficient participants within a given time frame in future larger RCT.

A key finding of the BRIGHT pilot trial is that there is little evidence to support the addition of breast cancer-tailored dietitian-led group support with WW referral due to its less promising outcomes, higher costs, as well as other implementation barriers suggested by the facilitators (i.e. lack of time and staff issues). The BRIGHT study has made an important contribution to the existing evidence of feasibility and pilot studies for weight loss in breast cancer patients. Evaluation of the BRIGHT trial process was useful for the purposes of assessing feasibility assessment and highlighting where potential threats to the internal and external validity of a large-scale RCT are likely to occur.

Our study shows promise, as over half of the participants in the WW group (58%) compared to 37% in the WW Plus group achieved a clinically important ≥ 5% loss of initial weight at 12 weeks. It is conceivable that more participants would have achieved clinically meaningful weight loss if the intervention was continued for longer such as up to 6 months or more [41].

At 12-month follow up, ≥ 40% of participants in all three groups achieved ≥ 5% weight loss. These findings are higher than the findings of Jolly et al.’s study (the Lighten Up trial) of a general population which involved eight programmes (WW; Slimming World; Rosemary Conley; group based, dietetics-led programme; general practice one-to-one counselling; pharmacy-led one-to-one counselling; choice of any of the six programmes and a comparator group), delivered for 12 weeks [32]. In that trial, the highest proportion of participants (31%) who sustained ≥ 5% weight loss from baseline at 12 months was from the WW group as compared to the other trial groups. Other RCTs of WW programmes with non-cancer patients have shown lesser weight loss-maintenance, although these interventions were of longer duration, compared to the BRIGHT trial [33, 42, 43]. A possible explanation is that BRIGHT trial participants were breast cancer survivors and hence, were more motivated to lose extra weight and maintain it to improve breast cancer-related prognosis. It is also possible that some participants had a recurrence of their cancer, and were unaware of this, which might have contributed to weight loss.

A study of breast cancer survivors in the USA (mean BMI 35.5 ± 3.9 kg/m^2^, mean age 51.7 ± 8.4 years), using either WW, intensive individual dietitian support for 12 months or both, found that participants receiving both interventions lost almost four times more weight than the group using WW alone at 12 months [34]. However, the extra support and 2 weeks longer intervention period had no impact on sustaining the weight loss in the WW Plus group after trial exit. Another randomised trial of overweight and obese adults in the USA (90% women, mean BMI 36.2 ± 5.5 kg/m^2^, mean age 49.7 ± 9.2 years) found that those receiving WW programme alone lost significantly more weight at 48 weeks than those receiving WW programme following group-based behavioural counselling [44]. Some participants reported that the transition to WW was difficult following the counselling due to differing approaches towards weight loss and changes in group size and leadership disrupting their weight loss progress [44]. This may indicate that the breast cancer-tailored dietitian-led support group sessions in our study reduced the likelihood of the participants adhering to the WW programme which could similarly be due to issues related to transition between groups. On the other hand, the WW only group followed one approach which may have led to improved weight loss maintenance.

Previous research suggested that group interventions for cancer patients are useful to reduce symptoms of depression and anxiety, improve social support and self-efficacy for coping, reduce pain and improve QoL among survivors [45, 46]. Our pilot study did not suggest any benefits of offering breast cancer-tailored dietitian-led group support in addition to WW referral. Psychological distress and depressive symptoms are typically highest in the first 6 months following cancer diagnosis and then usually decline over time as women adjust to the initial shock of diagnosis and acute effects of cancer treatment (Bower J.E. 2008 [62]). The rates of psychological symptoms of disease-free breast cancer survivors are comparable to women in the general population, although a subset of women may continue to experience negative symptoms for years after treatment (Bardwell, Wayne A 2006 [63]). Therefore, it is possible that as most participants joined the BRIGHT trial at least 1 year post-diagnosis, their psychological symptoms (e.g. feeling less optimism, having lower self-esteem) were less severe than closer to the diagnosis. Other studies suggest that health professional’s support and/or peer group support in cancer patients may add benefit, but this needs to be weighed against the costs depending on existing resources [34]. The WW Plus arm incurred higher cost in delivering this intervention compared to other arms due to the involvement of a dietitian and a breast care nurse. On the other hand, providing WW referral was inexpensive and effective for weight loss maintenance and improving QoL without the support of the breast cancer peer group and health professionals. Therefore, it has raised opportunities for further research, namely running of a definitive trial involving a WW group and a control for this patient group.

### Strengths and limitations

The strength of the study is that we have demonstrated feasibility of the trial procedures in a definitive trial and shown that there is little evidence to suggest proceeding with the WW Plus arm in a future definitive trial. Another strength of the study is that we did not use self-reported measures, except for three participants at 12 months follow-up who could not attend and hence sent by post, for weight changes to avoid the drawbacks of under-reporting for weight or BMI and over-reporting for height [47, 48].

A limitation of this study is that it was mainly conducted by one individual with great enthusiasm for the project, and results achieved in these types of trials might not give a true picture of a weight loss intervention in a multi-site trial or a real-world setting. This may be due to protocol adherence and enthusiasm in the trial participants and trial centres or because our participants were motivated to participate in a weight loss programme as invited by a breast consultant and/or the trial was funded by a cancer charity or for the good of others [49, 50]. Therefore, we recommend endorsement of the programme by a breast care consultant and breast cancer charity in future trials or during implementation. However, the participants seemed satisfied with the way in which the intervention was delivered; otherwise, it is unlikely that they would have continued and consequently, the intervention would have had a poor retention rate and been less likely to achieve any positive outcomes [51, 52]. Furthermore, few follow-up time points for outcome measures were used in this trial to reduce burden on participants and improve retention.

We were unable to recruit the targeted number of participants, due to limited time and resources available. In our trial, some ineligible women were invited because BMI was not recorded in medical records in outpatient clinics. Therefore, we recommend measuring and recording weight and BMI of breast cancer patients during every clinic visit should be performed to increase efficiency for future research. In addition, as body weight is an important prognostic factor, health professionals and researchers should have access to these data.

Secondary outcome data need to be interpreted with caution because this pilot stage was not fully powered to test for differences in outcomes (Shanyinde M. 2011 [58]). Adequately powered RCTs with long-term follow-up are needed to observe any differences in weight-loss and impact on survival. In future trials, we recommend randomisation, allocation and outcome assessment should be conducted by an independent person not involved in the trial design, recruitment and/or its delivery. The suggested costs of the programme were the direct costs of each intervention arm but we have not determined the cost to the participants of attending the programme or if they chose to purchase any products from the WW programme (they do not need to, but may have chosen to do so). In future trials, cost-effectiveness should be assessed and reported to enable the decision making process for future implementation of the programme.

### Possible next steps

In the future, a pragmatic single-blind multi-site weight loss maintenance trial should be considered based on the findings of the BRIGHT pilot trial and following discussions with experts in this area and various stakeholders including WW. Based on current findings, a hypothesis could be proposed as follows: a greater proportion of participants in the WW group will achieve ≥ 5% weight loss at 12 weeks and will maintain the weight loss at 15 months (or 12 months post-intervention) compared to the control. As a 12-week referral scheme is already in use by primary care in the UK, the authors suggest keeping the duration of the intervention as 12 weeks. The ultimate aim of most weight loss studies is to maintain initial weight loss over a long period of time (Borek, Aleksandra J 2015 [64]) ideally 12 months or longer [15] (SPH 2010 [65]). The WW programme is available in the community for weight loss and maintenance; therefore, the participants would be encouraged to follow the programme, attending group meetings and/or using the online resources. This would also measure the impact of continuation of attending WW meetings by participants’ own costs on sustainability of the trial (i.e. providing 12 week free access to WW meetings). Furthermore, the WW programme is suitable for a wide range of people with different cultural and/or dietary restrictions and therefore, no major adaptation would be required if participants from different ethnicity are to be recruited in the future RCT.

Participant-related outcomes would be measured at baseline, 3 months, 12 and 15 months post-randomisation.

Recruitment of sufficient participants in an efficient manner is still a major challenge to RCTs (Donovan, Jenny L 2016 [66]). To optimise recruitment and enrolment in the future, breast consultants or breast care nurses and cancer support centres from a number of sites could be involved as recruitment through oncologists’ office (33%) or cancer support centres (50%) has shown better enrolment of participants compared to mailing from research office (14% in the BRIGHT pilot trial) (Pinto Bernardine M 2004 [67]; Pakilit, Amber T 2001 [68]). Pro-active recruitment during clinic visits may also result in inviting only potentially eligible participants, lowering cost per enrolled participant (due to exclusion of staff time to assess eligibility and postal costs) and achieving a better response rate by gaining acceptance and trust from their health professionals for participating in a RCT (Samelson, E.J. 2008 [69]; Donovan, Jenny L 2016 [66]).

To demonstrate clinically meaningful 5% weight loss at 12 months, a range of sample sizes are suggested based on the BRIGHT pilot trial and another weight loss study, the Lighten-UP trial (targeted weight loss-maintenance using a range of commercial and primary care led programmes for 12 weeks in the UK and followed the participants for 1 year) [32] (Additional file 1: Appendix 4). These calculations reflect on the proportion of participants in the control and intervention arm achieving 5% weight loss maintenance at 12 months post-intervention follow-up (conducted by SF using nQuery Sample size calculator, and 90% power and 5% significance two-tailed test). As an example, using more realistic figures from the Lighten-Up trial compared with possibly overly optimistic higher values from our study, if 20% of the control group (17% in the Lighten-Up trial) and 50% of the intervention group (64% WW group in BRIGHT and 31% WW group in the Lighten-Up trial) achieve ≥ 5% weight loss, then 58 participants per group would be needed, so total *n* = 116. Assuming dropout would be 30%, then the total number recruited needs to be 116/0.7 = 166, which is *n* = 83 per group. The sample size will inflate if the control group success rate is higher and if the difference between control and intervention is smaller.

## Conclusions

The BRIGHT trial procedures were feasible, with minor modifications. This pilot trial indicates the benefits of providing free WW vouchers for weight loss maintenance and improving QoL but provided no evidence that including a dietitian-led group support as delivered in this study would add any extra value. Further research on providing free WW vouchers with long-term (≥ 10 years) follow-up should be assessed for its sustainability on weight loss maintenance, benefit on survival and other health outcomes in women treated for breast cancer.

## Additional file

Additional file 1: **Appendix 1.** Summary of preliminary study findings. **Appendix 2.** Contents of the dietitian led support group meetings of the WW Plus group. Appendix 3. Intervention Fidelity Checklist. **Appendix 4.** Sample size estimation. (DOCX 55 kb)

## Abbreviations

ARI: Aberdeen Royal Infirmary
BCT: Behaviour change techniques
BMI: Body mass index
FACT-B: The Functional Assessment of Cancer Therapy-Breast
MRC: Medical Research Council
NICE: The National Institute for Health and Care Excellence
QoL: Quality of life
RCT: Randomised controlled trial
UK: United Kingdom
WW: Weight Watchers
