# Supplementary material for: Pilot randomised controlled trial of Weight Watchers® referral with or without dietitian-led group support for weight loss in women treated for breast cancer: the BRIGHT (BReast cancer weIGHT loss) trial
Source: Pilot Feasibility Stud. 2019 Feb 13;5:24. doi: 10.1186/s40814-019-0405-x (PMC6373055; doi:10.1186/s40814-019-0405-x)
Supplement: Supplementary file 1 — Appendix 1. Summary of preliminary study findings. Appendix 2. Contents of the dietitian led support group meetings of the WW Plus group. Appendix 3. Intervention Fidelity Checklist. Appendix 4. Sample size estimation. (DOCX 55 kb) [file 40814_2019_405_MOESM1_ESM.docx]

**Additional file 1: Summary of preliminary study findings**

Table A: Setting, format, intensity and provider

| ***Intervention design feature*** | ***Systematic review*** | ***Guidelines (adult population with or without cancer)*** | ***Mixed methods study*** |
| --- | --- | --- | --- |
| ***Setting*** | Community/home/clinic/community/  hospital | Community/home/religious-based/workplace/internet or web/mailed print materials/telephone/ primary care [1-3]. | A relaxed non-hospital setting such as a cancer support centre or a community centre. |
| ***Format:***  ***information session*** | Individual contact^*^ by telephone or face-to-face/group based sessions^*^/combination^*^ of group and individual contacts | Individual contact/group based sessions [3] and/mixed [2, 4]. | A small group of women treated for breast cancer. |
| ***Format:***  ***Physical activity*** | Individual/group sessions^*^(supervised/unsupervised) | Individual/group sessions (supervised/unsupervised) /combination of both [4]. | Mixed views:  Performing on their own,  activities in a group with other women treated for breast cancer. |
| ***Suitable time to join a programme*** | ≥ 6 month post-treatment/4-14 years post-diagnosis.  No clear evidence on best time to join. | After completing surgery, chemotherapy and/radiation therapy [5, 6]/soon after diagnosis/anytime/ when participants are physically and psychologically prepared and motivated [1, 5]. | A weight loss programme can be offered to all after completing treatments preferably 3-6 months post-treatment. |
| ***Who delivers the intervention*** | Dietitian/nutritionist/research staff/dietary counsellors/commercial weight management group leaders  No clear association was found between provider and effectiveness of interventions | Doctors/dietitian/psychologist/nurses/exercise specialist/lay person [2, 3].  Certified exercise specialist/ physical therapist with knowledge of cancer related side -effects and late effects [7, 8] | A trained person with the knowledge of diet, weight loss and cancer.  A dietitian or a breast care nurse.  A physiotherapist or an exercise expert to conduct the exercise components. |
| ***Frequency of contact/group session/duration of the programme*** | No clear association was found between frequency of contacts made with participants or duration of programme and effectiveness of interventions. | Widely varied [4] but longer duration or frequent contacts are found to be effective than shorter duration or less contacts with the participants [2]. | Preferred duration (3-6 months) and frequency of contact (weekly to every two months) widely varied. |

*^*= Significant weight loss achieved by the intervention component and BCT= Behaviour change techniques^*

Table B: Components of a weight loss programme

| ***Intervention design feature*** | ***Systematic review findings*** | ***Guidelines (adult population with or without cancer*** | ***Mixed methods study findings*** |
| --- | --- | --- | --- |
| **Components of a weight loss programme** | Diet alone^*^  Diet and physical activity^*^  Diet, physical activity and BCT^*^  Weight Watchers® programme^*^ | A combination of diet, physical and behavioural therapy in adult population [3, 9].  Diet and physical activity [4, 6] and behavioural strategies for weight loss in cancer survivors [10].  Commercial weight management programmes^:^ Weight Watchers® programme [3, 9, 11]. | A combination of dietary, physical activity, knowledge exchange and hands on session.  A commercial weight management programme for its flexibility and motivation. |
| **Dietary components** | Low fat^*^/reduced energy^*^ | Reduced energy/low calorie [3, 9, 12].  Eat a variety of healthy foods from plant sources and limit the amount of processed meat or red meat [10, 13]. | Information was preferred on types/amount of food/how often should be eaten.  Encouragement to eat healthier. |
| **Physical activity** | Circuit followed by stretching^*^/ demonstration of muscle strengthening exercise^*^/guidance and motivation for performing regular exercise^*^. | Guidance/prescribe (e.g. 45/60 minutes five times/week [9])  For cancer survivors activity should be prescribed depends on individual ability and need [4, 6, 14]. | Exercise session and/or information and advice on suitable physical activities.  Mostly preferred physical activity was walking |
| **Other** | Social support.  Advices on shopping/food choices (e.g. eating out)/smart snacking/energy balance /hydration.  Cooking guide or classes  BCTs used: goal setting, self-assessment, problem solving, motivational support. | Social support [2, 11, 15, 16]. Advice on limiting portion size, healthy snacking and checking food labels [6, 17].  BCTs commonly used: providing feedback on performance, goal review [2, 18]/goal setting; action planning; barrier identification and/or problem solving; graded tasks; self-monitoring of behaviour; instruction on how to perform behaviour; and planning social support and/or social change [18]. | Encouragement to make friends from the group for social support  Measuring body weight as part of the programme.  N/A (a psychologist was not part of the study team, therefore, BCTs were not explored in this stage). |

*^*= Significant weight loss achieved by the intervention component and BCT= Behaviour change techniques.^*

References

1. Stull VB, Snyder DC, Demark-Wahnefried W: Lifestyle interventions in cancer survivors: designing programs that meet the needs of this vulnerable and growing population. *J Nutr* 2007, 137(1 Suppl):243S-248S.

2. Greaves CJ, Sheppard KE, Abraham C, Hardeman W, Roden M, Evans PH, Schwarz P, IMAGE Study Group: Systematic review of reviews of intervention components associated with increased effectiveness in dietary and physical activity interventions. *BMC Public Health* 2011, 11:119-2458-11-119.

3. NICE: Obesity:the prevention, identification, assessment and management of overweight and obesity in adults and children. 2006, .

4. Pekmezi DW, Demark-Wahnefried W: Updated evidence in support of diet and exercise interventions in cancer survivors. *Acta Oncol* 2011, 50(2):167-178.

5. Spark LC, Reeves MM, Fjeldsoe BS, Eakin EG: Physical activity and/or dietary interventions in breast cancer survivors: a systematic review of the maintenance of outcomes. *Journal of Cancer Survivorship* 2013, **7**(1):74-82.

6. Rock CL, Doyle C, Demark‐Wahnefried W, Meyerhardt J, Courneya KS, Schwartz AL, Bandera EV, Hamilton KK, Grant B, McCullough M: Nutrition and physical activity guidelines for cancer survivors. *CA: a cancer journal for clinicians* 2012, 62(4):242-274.

7. ACSM experts: American College of Sports Medicine roundtable on exercise guidelines for cancer survivors. 2010, .

8. Wolin KY, Schwartz AL, Matthews CE, Courneya KS, Schmitz KH: Implementing the exercise guidelines for cancer survivors. *J Support Oncol* 2012, 10(5):171-177.

9. SIGN: Scottish Intercollegiate Guidelines Network: Management of obesity: a national clinical guideline. *Scottish Intercollegiate Guidelines Network: Edinburgh* 2010, **115**.

10. ACS: Lifestyle changes that make a difference. 2012, .

11. Jolly K, Lewis A, Beach J, Denley J, Adab P, Deeks JJ, Daley A, Aveyard P: Comparison of range of commercial or primary care led weight reduction programmes with minimal intervention control for weight loss in obesity: lighten Up randomised controlled trial. *BMJ* 2011, 343:d6500.

12. NICE: Obesity: identification, assessment and management of overweight and obesity in children, young people and adults. 2014, 189.

13. WCRF/AICR: Food, nutrition, physical activity, and the prevention of cancer: A Global Perspective Expert Report. 2007, .

14. Panel E: American College of Sports Medicine roundtable on exercise guidelines for cancer survivors. 2010, :1409-14026.

15. Hoddinott P, Allan K, Avenell A, Britten J: Group interventions to improve health outcomes: a framework for their design and delivery. *BMC Public Health* 2010, **10**:800-2458-10-800.

16. Sammarco A: Perceived social support, uncertainty, and quality of life of younger breast cancer survivors. *Cancer Nurs* 2001, 24(3):212-219.

17. Cancer Research UK. How to keep a healthy weight [<http://www.cancerresearchuk.org/cancer-info/healthyliving/obesity-bodyweight-and-cancer/ten-top-tips/ten-top-tips-weight-loss-tips-based-on-scientific-evidence>] Assessed 5 Nov 2017

18. Hartmann-Boyce J: How components of behavioural weight management programmes affect weight change. 2013, Review 1b.

**Appendix 2 Table: Contents of the dietitian led support group meetings of the WW Plus group**

| Meeting | Delivered by | Topics discussed |
| --- | --- | --- |
| Week 1: Group meeting 1 | Dietitian | Food groups, healthy food choices, the Eatwell plate, fluid/ salt/ alcohol intake, fad diets, healthy weight, myths about diet and cancer, introduction to WW. |
| Week 2: Group meeting 2  (Participants received WW vouchers) | **First session**: Breast care specialist nurse  **Second session**: Dietitian | **First session**: reasons behind weight gain, altered body image, relationship issues, tips for maintaining a positive body image, prosthesis/ underwear and available supports.  **Second session**: evidence related to cancer and physical activity, types/ amount and intensity of suitable physical activities, tips for being more active. |
| Week 6: Group meeting 3  (week 4 of WW intervention) | Dietitian | Food labels, recommended portion sizes, calorie counting and tips for portion control. |
| Week 10: Group meeting 4  (week 8 of WW intervention) | Dietitian | Group discussion of food and drink models showing amounts of sugar and fat in common foods and takeaway meals. |
| Week 14: Group meeting 5  (week 12 of WW intervention) | Dietitian | Overview of the previous meetings followed by a diet-quiz fun session with prize distribution to the winners. This meeting consolidated what they had learnt over the course of the intervention. |

**Appendix 3:** **Intervention Fidelity Checklist**

Adherence to content and competence in delivering the intervention

| **Checklist** | **Rating** |
| --- | --- |
| Leader correctly coveys and communicates programme principles | Yes/No |
| Leader effectively uses role-play to teach a principle or strategy e.g. label reading | Yes/No |
| Participants have the chance to ask questions | Yes/No |
| Leaders effectively responds when participants ask questions | Yes/No |
| Length of the session 1-1.5 hour | Yes/No |
| Total number of contacts per batch, n=5 sessions | Yes/No |
| Length of intervention period, 14 months | Yes/No |
| Leader adhered to the contents of the session as planned | Yes/No |

**Appendix 4: Sample size estimation**

|  | **Intervention** | |  |  |  |  |  |  |  |  |  |  |
| --- | --- | --- | --- | --- | --- | --- | --- | --- | --- | --- | --- | --- |
| **Control** | **10** | **15** | **20** | **25** | **30** |  | **35** | **40** | **45** | **50** | **55** | **60** |
| **5** | 621 | 207 | 114 | 75 | 55 |  | 42 | 34 | 28 | 23 | 20 | 17 |
| **10** | - | 957 | 286 | 146 | 92 |  | 65 | 49 | 38 | 31 | 25 | 21 |
| **15** | - | - | 1252 | 354 | 174 |  | 107 | 73 | 54 | 42 | 33 | 27 |
| **20** | - | - | - | 1504 | 412 |  | 198 | 119 | 80 | 58 | 44 | 35 |
| **25** | - | - | - | - | 1714 |  | 460 | 216 | 128 | 85 | 61 | 46 |
| **30** | - | - | - | - | - |  | 1882 | 496 | 230 | 134 | 88 | 63 |
| **35** | - | - | - | - | - |  | - | 2008 | 523 | 240 | 138 | 90 |
| **40** | - | - | - | - | - |  | - | - | 2092 | 538 | 244 | 140 |
| **45** | - | - | - | - | - |  | - | - | - | 2134 | 544 | 244 |
| **50** | - | - | - | - | - |  | - | - | - | - | 2134 | 538 |
|  |  |  |  |  |  |  |  |  |  |  |  |  |

*Note: Column and rows reflect percentage of participants achieving 5% weight loss-maintenance at 15 months follow-up period in the intervention and control arms, respectively.*
